# The Association Between Amino Acids and the Onset and Progression of Type 2 Diabetes Mellitus: A Comprehensive Analysis Based on UK Biobank Database

**DOI:** 10.1155/jdr/8033429

**Published:** 2026-01-12

**Authors:** Jiayu Yang, Zelong Wu, Jike Fang, Zhongyan Zhang, Jiangyuan Huang, Yajie Wang, Jinwei Cui, Shiye Ruan, Qian Yan, Qianlong Wu, Sheng Chen, Baohua Hou, Shanzhou Huang, Chuanzhao Zhang

**Affiliations:** ^1^ Department of General Surgery, Guangdong Cardiovascular Institute, Guangzhou, China; ^2^ Department of General Surgery, Guangdong Provincial People’s Hospital (Guangdong Academy of Medical Sciences), Southern Medical University, Guangzhou, China, fimmu.com; ^3^ Department of Hepatobiliary Surgery, Maoming People’s Hospital, Maoming, China; ^4^ The Second School of Clinical Medicine, Southern Medical University, Guangzhou, China, fimmu.com; ^5^ South China University of Technology School of Medicine, Guangzhou, China; ^6^ Department of General Surgery, Heyuan Key Laboratory of Molecular Diagnosis & Disease Prevention and Treatment, Heyuan People’s Hospital, Heyuan, Guangdong Province, China

**Keywords:** amino acids, Mendelian randomization, Type 2 diabetes, UK Biobank

## Abstract

**Background:**

Recent studies have demonstrated an association between amino acids (AAs) and the occurrence of Type 2 diabetes mellitus (T2DM). However, whether there is an underlying causal relationship between AAs and T2DM, as well as their potential links to T2DM progression, complications, and treatment selection, still lacks sufficient clinical evidence.

**Methods:**

This study included 205,208 participants from the UKB database, with 14,066 diagnosed with T2DM. We used LASSO regression, supplemented by Mendelian randomization (MR), to explore the causal relationship between AA levels and T2DM. Additionally, restricted cubic splines, receiver operating characteristic (ROC) curves, and multivariable‐adjusted regression models were applied to analyze the association between AA levels, insulin resistance, secondary complications, and treatment options for T2DM.

**Results:**

Alanine and valine were positively associated with T2DM, while glutamine, glycine, and histidine were negatively associated with T2DM. In particular, the MR results indicated a causal relationship between T2DM and plasma glutamine and glycine, which were identified as protective factors. Other branched‐chain AAs, such as leucine and isoleucine, did not show significant positive associations in the regression analysis. Additionally, we integrated various AAs to develop a predictive model for secondary complications of T2DM. The model demonstrated high predictive accuracy for a range of T2DM‐related complications (all areas under the ROC curve > 0.730). In addition, specific AA profiles were related to insulin resistance and demand for insulin or oral hypoglycemic drug treatment.

**Conclusion:**

Our study results demonstrate a close relationship between AA levels and the occurrence, development, and treatment demand for T2DM, offering potential biomarkers for predicting complications and guiding personalized treatment.

## 1. Introduction

Type 2 diabetes mellitus (T2DM) represents a significant and escalating global health crisis, with the World Health Organization (WHO) estimating that diabetes impacts over 9% of the world’s adult population, with T2DM constituting approximately over 90% of these cases [1, 2]. T2DM not only leads to abnormalities in glucose metabolism but also imposes a significant healthcare burden due to its multisystem and multiorgan complications. These complications include macrovascular diseases (such as cardiovascular disease and stroke) [3, 4], microvascular complications (such as diabetic nephropathy, retinopathy, and neuropathy), neurological damage, and an increased risk of certain cancers [5, 6]. The complexity and diversity of these secondary conditions collectively contribute to markedly increased morbidity and mortality among T2DM patients, placing substantial strain on both patients’ quality of life and healthcare systems. Despite considerable research efforts, a comprehensive understanding of the pathogenesis of T2DM remains elusive. Traditional risk factors, including obesity, sedentary lifestyle, and genetic predisposition, are well‐documented [7–10]. However, emerging evidence suggests that metabolic dysregulation of amino acids (AAs) plays a pivotal role in the development and progression of T2DM [11–13]. AAs are not only essential building blocks of proteins but also serve as key regulators of metabolic pathways and signaling molecules. Their metabolism is intricately linked to glucose homeostasis and insulin sensitivity [14], yet this connection is often overlooked in mainstream diabetes research.

In the realm of existing research, the correlation between AAs and T2DM has been widely confirmed. Several reliable studies have shown that elevated plasma levels of branched‐chain amino acids (BCAAs) are positively associated with an increased risk of T2DM [15–17]. A robust and consistent body of evidence has established a positive correlation between elevated serum concentrations of BCAAs and the risk of T2DM development. The pathophysiological underpinning of this association is postulated to involve activation of the mammalian target of rapamycin complex 1 (mTORC1) [18]. Furthermore, several prospective studies have indicated that variations in plasma levels of aromatic AAs—phenylalanine, tryptophan, and histidine—as well as glycine, are associated with the onset of T2DM [19–21].

Despite these findings, current literature predominantly mainly highlights the role of AAs as diagnostic biomarkers for T2DM. And the preponderance of evidence is derived from a multitude of small‐scale, prospective investigations, which may be susceptible to limitations in methodological rigor and demographic representativeness. Additionally, T2DM may be accompanied by severe complications, such as diabetic nephropathy and diabetic retinopathy, which often signify that it has advanced to a later stage, associated with considerable rates of disability and mortality [22]. However, the role of AAs in predicting these complications has not yet been adequately confirmed in clinical research. The therapeutic implications of AA levels in T2DM management are also a matter of ongoing debate. The capacity for AA levels to inform treatment decisions, including the selection between insulin therapy and oral hypoglycemic agents, represents a promising area for future research. In summary, direct clinical evidence illustrating the potential of AAs in the diagnosis, complication prediction, and treatment of diabetes is currently limited.

The United Kingdom Biobank (UKB) database, an extensive repository of genetic, clinical, and lifestyle data from over half a million participants, presents a unique opportunity to explore the relationship between AA levels and the occurrence, progression, and treatment of T2DM within a large and diverse population. Prior studies utilizing the UKB database have identified novel genetic and environmental risk factors for T2DM, yet the role of AAs in T2DM development within this context has not been exhaustively explored [23].

This prospective study is aimed at elucidating the interrelationship between AAs and T2DM from both real‐world research and genetic perspectives by utilizing the UKB database and employing a two‐sample Mendelian randomization (MR) approach. Comprehending the association between AAs and T2DM is essential for the advancement of personalized clinical strategies, the enhancement of early detection mechanisms, and the formulation of targeted preventive measures. This understanding is conducive to the development of tailored nutritional guidance and innovative therapeutic approaches that directly address the metabolic determinants of diabetes risk. It also amplifies the impact of public health initiatives, contributes to economic savings by alleviating the diabetes burden on healthcare systems, and bolsters global endeavors to counter this prevalent disease.

## 2. Method

### 2.1. Data Source

The data for this study were extracted from the UKB database (https://www.ukbiobank.ac.uk/), a comprehensive prospective database that is aimed at examining the effects of genetic, environmental, and lifestyle factors on the spectrum of diseases. The UKB has gathered extensive health‐related information from approximately 500,000 volunteers throughout the UK, including genotypic data, biomarkers, lifestyle habits, and medical histories. Our analysis focused on a subset of the UKB cohort, comprising 205,208 participants, of which 14,066 had a diagnosis of T2DM. The dataset used was accessed on May 7, 2024, based on the version released as of December 9, 2023. The correlation between AA levels and T2DM was explored, with further evaluation of their potential implication in disease management undertaken.

We utilized summary statistics from genome‐wide association studies (GWAS) to conduct a two‐sample MR study on the causal relationship between AAs and T2DM; exposure factors were obtained from the “plasma metabolome GWAS atlas,” which prioritizes metabolites influencing a range of human diseases, including comprehensive GWAS for AAs. Outcome factors were derived from data consolidated from three renowned cohorts: the Finngen GWAS for T2DM (finngen_R11_T2D), the UKB’s SAIGE project (ukb‐saige‐250.24), and the EU‐led consortium led by Imperial College London (ieu‐a‐1090). Detailed information on the GWAS data sources is presented in Supporting Information (available here). These cohorts, with their extensive genetic and phenotypic data, have been instrumental in our efforts to understand the genetic susceptibility to T2DM.

### 2.2. Study Population

We applied a set of stringent inclusion criteria. We focused on adults, ensuring that participants were over the age of 18, thereby circumventing the developmental and metabolic variation characteristic of pediatric populations. We meticulously excluded individuals with a prediagnosis of T2DM to examine the incipient relationships between AA levels and the subsequent development of the disease. The inclusion of only those with complete AA data guaranteed the integrity and reliability of our exposure assessment, facilitating a robust statistical evaluation. Furthermore, by excluding participants with a history of malignant tumors, we mitigated the potential confounding effects that oncological conditions and their treatments could impose on metabolic profiles.

### 2.3. Definition

In our study, T2DM is operationally defined according to the International Classification of Diseases, 10th Revision (ICD‐10) criteria, which categorizes the condition under the code E11 for non–insulin‐dependent diabetes mellitus [24]. The ICD‐10 framework serves as the primary diagnostic benchmark, with additional corroborative evidence drawn from patients’ medical histories, including documented hyperglycemia, and pharmacological data, such as the use of oral hypoglycemic agents or insulin therapy. The use of insulin is primarily determined based on relevant questionnaires, while the definition of oral hypoglycemic agents mainly refers to the use of metformin, gliclazide, repaglinide, and acarbose. Diabetes‐related complications are also defined according to the ICD‐10 classification. Insulin resistance (IR) levels are calculated using the estimated glucose disposal rate (eGDR) formula: eGDR = 21.158 − [0.09 × waist circumference (WC, cm)] − [3.407 × hypertension (yes/no)] − [0.551 × hemoglobin A1c (HbA1c, *%*)]. Based on the eGDR score quartile definition, if the score is below the 25th percentile (P25), it is defined as IR.

### 2.4. Study Design

**Figure 1:**
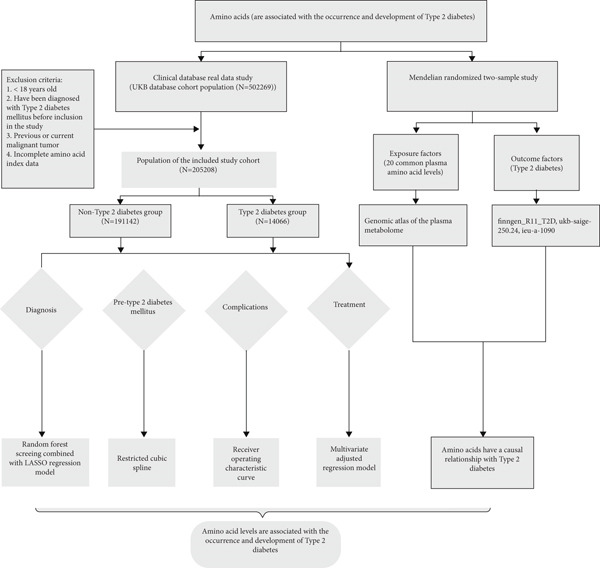
Flowchart of the research process.

This research utilized data for nine distinct AAs from the UKB dataset to investigate their association with T2DM using ;least absolute shrinkage and selection operator (LASSO) logistic regression analysis. Building on these findings, we employed a two‐sample MR approach to explore potential causal relationships between 20 AAs and T2DM. To further elucidate the relationship between AA metabolism and T2DM complications, as well as IR, we performed a range of statistical analyses. receiver operating characteristic (ROC) curves were used to evaluate the prognostic value of AA levels as biomarkers for T2DM‐related complications. Additionally, we utilized restricted cubic spline (RCS) to define the nonlinear relationships between AA levels and the risk of T2DM complications. Logistic regression models were also adjusted for multiple covariates to assess the relationship between specific AA levels and the likelihood of insulin dependence (Figure 1).

### 2.5. Statistical Analysis

**Figure 2:**
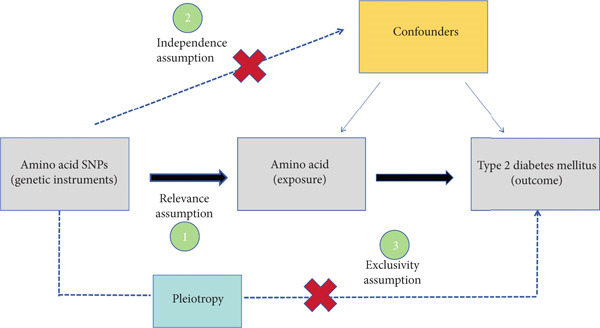
Mendelian randomization study process. By leveraging the random allocation of alleles at conception, MR mimics a randomized controlled trial, reducing confounding and reverse causation. This approach relies on three key assumptions: The genetic variants must be associated with the 20 common AAs (relevance), independent of confounders (independence), and influence T2DM only through the amino acids (exclusion restriction). If these assumptions hold, MR allows for robust causal inference between the exposure and the outcome.

In this study, continuous variables at baseline are presented as medians, while categorical variables are expressed as frequencies with proportions. We employed *t*‐tests, analysis of variance, or chi‐square tests to assess differences between groups. For the two‐sample MR analysis, the primary exposures were 20 common AAs, and their genetic instruments were single nucleotide polymorphisms (SNPs). SNPs were selected based on the following criteria: (1) genome‐wide significance (*p* < 5 × 10^−8^); (2) independence, defined as linkage disequilibrium (LD) *r* ^2^ < 0.001 and physical distance > 10,000 kb; and (3) an *F*‐statistic > 10 to avoid weak instrument bias. The *F*‐statistic was calculated as *F* = *β* ^2^/SE^2^, and SNPs with *F* < 10 were excluded. Prior to conducting statistical analyses, summary statistics were harmonized according to previously recommended methods. Random‐effects inverse‐variance weighting (IVW) was applied, assuming all instrumental variables are valid. Due to the potential for directional pleiotropy bias with the aforementioned method, we conducted sensitivity analyses for the two‐sample MR (weighted median method, MR‐Egger method, and MR‐PRESSO method) to test the reliability and stability of the main MR assumptions. The causal effect was considered robust when at least three MR methods yielded similar estimates. Additionally, we used Cochran’s *Q* test to check for heterogeneity. To determine if a single SNP was driving the causal association, leave‐one‐out sensitivity analyses were performed by sequentially removing individual variants from the analysis (Figure 2). All major analyses were performed using R Version 4.3.2. The following R packages were used: glmnet for LASSO regression; TwoSampleMR and MRPRESSO for MR analysis; rms and splines for RCS modeling; randomForest for variable importance; pROC for ROC curve analysis; stats for multivariable logistic regression; ggplot2, forestplot, and gridExtra for visualization; and dplyr, tidyr, readxl, and data. table for data processing. Descriptive statistics and basic data cleaning were performed in SPSS Version 27.0. The data analysis was carried out between June and July 2024. The results of the logistic regression are presented as odds ratios with 95% confidence intervals, and *p* values less than 0.05 were considered to indicate statistical significance.

## 3. Results

### 3.1. Demographic Information and AA Levels Between Patients With and Without T2DM

Patients with T2DM exhibit significant differences from nondiabetic individuals in demographic information and AA levels. The study results in Table 1 indicate that T2DM patients are older, with an average age of 60.00 years, compared to the control group with an average age of 57.00 years (*p* < 0.001). A higher body mass index (BMI) is observed in T2DM patients, with a median of 30.70, as opposed to 26.50 in the nondiabetic group (*p* < 0.001). The prevalence of male gender (59.4%) and smoking (53.5%) is more common in the T2DM group. The differences in AA levels are also pronounced between the two groups. Compared to the nondiabetic population, the median levels of alanine, leucine, isoleucine, valine, and phenylalanine are elevated in the diabetic population. Conversely, the median levels of glycine, histidine, and glutamine were decreased, and these differences are statistically significant (*p* < 0.05).

**Table 1:** Demographics of patients with Type 2 diabetes mellitus.

**Variable**	**Type 2 diabetes mellitus**		**p** **value**
	**No,** **N** = 191,142^a^	**Yes,** **N** = 14,066	
Age (years)	57.00 (49.00, 62.00)	60.00 (53.00, 65.00)	< 0.001^b^
Body mass index	26.50 (24.00, 29.50)	30.70 (27.60, 34.60)	< 0.001^b^
Sex			< 0.001^c^
No	106,242 (55.6%)	5710 (40.6%)	
Yes	84,900 (44.4%)	8356 (59.4%)	
Smoking			< 0.001^c^
No	108,230 (56.9%)	6487 (46.5%)	
Yes	82,045 (43.1%)	7462 (53.5%)	
Alcohol			< 0.001^c^
No	7744 (4.1%)	1202 (8.6%)	
Yes	182,956 (95.9%)	12,809 (91.4%)	
Hypertension			< 0.001^c^
No	158,108 (82.7%)	8348 (59.3%)	
Yes	33,034 (17.3%)	5718 (40.7%)	
Amino acid			
Alanine (mmol/L)	0.286 (0.238, 0.341)	0.318 (0.265, 0.379)	< 0.001^b^
Glutamine (mmol/L)	0.554 (0.500, 0.608)	0.533 (0.474, 0.591)	< 0.001^b^
Glycine (mmol/L)	0.161 (0.130, 0.205)	0.139 (0.112, 0.172)	< 0.001^b^
Histidine (mmol/L)	0.065 (0.059, 0.072)	0.064 (0.057, 0.071)	< 0.001^b^
Isoleucine (mmol/L)	0.048 (0.039, 0.059)	0.054 (0.045, 0.067)	< 0.001^b^
Leucine (mmol/L)	0099 (0.084, 0.118)	0.111 (0.094, 0.131)	< 0.001^b^
Phenylalanine (mmol/L)	0.046 (0.040, 0.053)	0.049 (0.042, 0.056)	< 0.001^b^
Tyrosine (mmol/L)	0.061 (0.053, 0.070)	0.066 (0.057, 0.076)	< 0.001^b^
Valine (mmol/L)	0.204 (0.179, 0.232)	0.227 (0.200, 0.259)	< 0.001^b^

^a^Median (QR); *n* (%).

^b^Wilcoxon rank‐sum test.

^c^Pearson’s chi‐squared test.

### 3.2. Correlation of AA Levels With T2DM

To provide a more intuitive view of the relationships between various factors and the development of T2DM, we employed a random forest model. Two key metrics—mean decrease accuracy (MDA) and mean decrease Gini (MDG)—were used to assess variable importance. MDA reflects the impact of individual variables on prediction accuracy, with higher values indicating a greater contribution to the model’s overall accuracy. MDG measures how well a variable splits data within decision trees, where higher values represent stronger discriminatory power. In our analysis, most AAs exhibited significant importance for T2DM development, with MDA values exceeding 50 (except for glycine). Notably, tyrosine (MDA: 140.61), phenylalanine (MDA: 107.75), and valine (MDA: 98) ranked as the top three AAs, highlighting their strong association with T2DM risk. In contrast, age, sex, and smoking had lower MDA values, suggesting that they contribute less to T2DM risk prediction. When considering MDG, all AAs had values above 2000, indicating a robust ability to differentiate between diabetic and nondiabetic individuals. Among non‐AA factors, only BMI (MDG = 2987.29) showed substantial discriminative capacity, while age, sex, smoking, alcohol consumption, and hypertension had relatively smaller MDG values (Figure 3a and Table S1). Using LASSO regression, we eliminated multicollinearity among independent variables and identified factors associated with the development of T2DM. The results showed that specific AA levels were closely related to T2DM occurrence. Alanine (OR: 1.34, 95% CI: 1.32–1.37, *p* < 0.001) and valine (OR: 1.29, 95% CI: 1.27–1.32, *p* < 0.001) were identified as risk factors for T2DM, indicating that higher levels may increase the risk of developing the disease. In contrast, glutamine (OR: 0.85, 95% CI: 0.83–0.87, *p* < 0.001), glycine (OR: 0.78, 95% CI: 0.76–0.80, *p* < 0.001), and histidine (OR: 0.90, 95% CI: 0.88–0.92, *p* < 0.001) were found to be protective factors, potentially reducing the risk of T2DM to varying degrees. Additionally, leucine, isoleucine, tyrosine, and phenylalanine did not show a clear association with T2DM development. Interestingly, a history of alcohol consumption (OR: 0.41, 95% CI: 0.38–0.44, *p* < 0.001) was significantly associated with a lower risk of T2DM. On the other hand, baseline factors such as age, gender, smoking, hypertension history, and BMI were positively associated with the risk of developing T2DM (Figure 3b).

Figure 3Association between various influencing factors and T2DM. (a) Random forest algorithm model results. MDA and MDG are two commonly used feature importance evaluation indicators. MDA evaluates the importance of features by measuring the prediction accuracy of the random forest model. Specifically, it reflects the contribution of a feature to the model by randomly shuffling the value of a feature and calculating the change in accuracy. The greater the difference, the more important the feature. MDG is based on the Gini coefficient (or Gini impurity) to evaluate the ability of features to reduce impurity during tree construction. Each time a feature is used for node splitting, its contribution is recorded, and the same features in all trees are summarized, and the score is calculated. The greater the difference, the stronger the ability of the feature to identify different populations. MDA, mean decrease accuracy; MDG, mean decrease Gini. (b) Results of LASSO regression. OR, odds ratio; CI, confidence interval.

**Figure:**
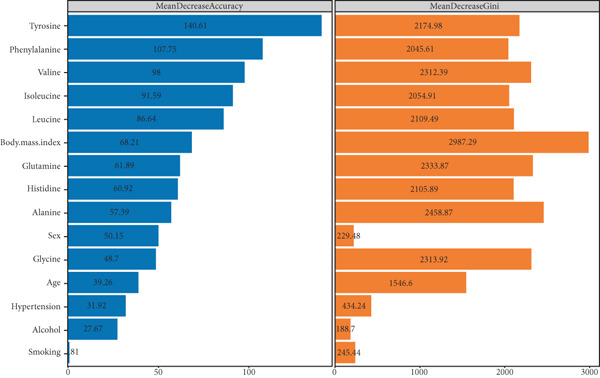
(a)

**Figure:**
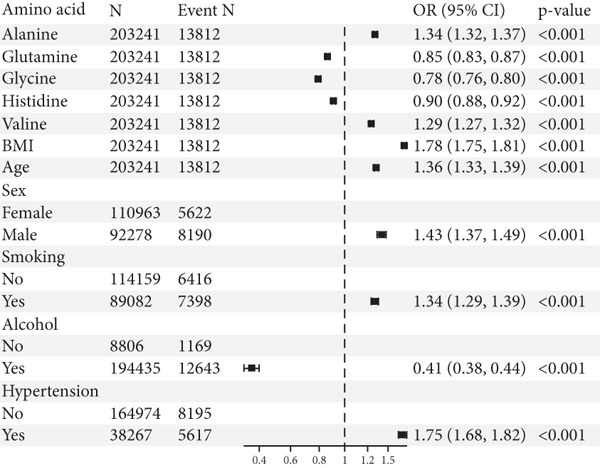
(b)

### 3.3. MR Investigates the Correlation Between Plasma AA Concentrations and the Risk of Developing T2DM

**Figure 4:**
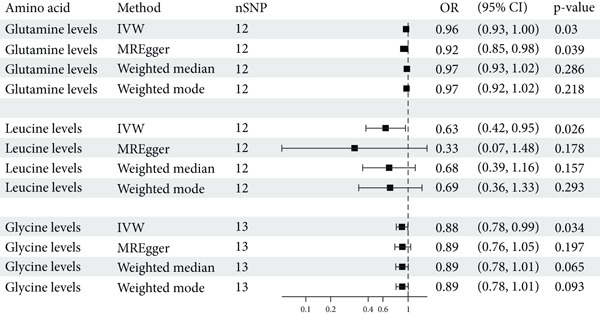
Positive results of a two‐sample MR study on 20 common AAs and T2DM. IVW, inverse variance weighted; OR, odds ratio; CI, confidence interval.

In this study, we conducted a two‐sample MR analysis using 20 common plasma AAs as exposure factors and T2DM as the outcome. We elected the IVW method as our primary analytical approach, while alternative methods, such as MR‐Egger, weighted median, and weighted mode, also provided valuable insights. Figure 4 presents the findings from our study, employing the IVW method, which suggests a causal association between plasma levels of glutamine (OR = 0.96, 95% CI: 0.93, 1.00, *p* = 0.030) and glycine (OR = 0.88, 95% CI: 0.78, 0.99, *p* = 0.034) with the incidence of T2DM. These results are in concordance with the analytical outcomes derived from the UKB database. In contrast, alanine, valine, and histidine were not found to be causally linked to T2DM. Notably, leucine is identified as a protective factor against T2DM, with a significant causal relationship (OR = 0.63, 95% CI: 0.42, 0.95, *p* = 0.026). Furthermore, other AAs, including aromatic AAs and methionine, were not determined to have a causal relationship with the development of T2DM.

To further validate the robustness of these findings, we constructed a scatter plot, funnel plot, and forest plot and performed leave‐one‐out analyses (Figure S1). The forest plots displayed the effect estimates for each SNP in the IVW analysis, while the scatter plots visually represented the estimates of the effects of each SNP on AAs and T2DM. These funnel plots revealed a certain degree of heterogeneity, which may indicate the diverse impacts of the selected SNPs used to represent AA levels (genetic instruments) on disease outcome. However, through MR‐Egger regression analysis, we found no strong evidence of horizontal pleiotropy, suggesting that genetic variants primarily influence outcomes through AAs rather than through other confounding biological pathways. The leave‐one‐out analysis, which involved excluding each SNP one by one and recalculating the estimates, further confirmed that our main findings were not driven by any single SNP, lending support to the robustness of our analysis.

### 3.4. Demographic Information, AA Levels, and Related Complications in Patients With T2DM

In a cohort of 14,066 patients diagnosed with T2DM, demographic stratification revealed a median age of 60 years (interquartile range [IQR]: 53–65). Among these individuals, 46.5% were female, and 53.5% were male. The prevalence of smoking was notable, with 53.5% of participants identified as smokers, while a significant majority, 91.4%, reported alcohol consumption. Hypertension was present in 40.7% of the cohort, accompanied by a median BMI of 30.7 (IQR: 27.6–34.6). AA levels, assessed through nuclear magnetic resonance, exhibited the following concentrations: alanine at 0.318 mmol/L (IQR: 0.265–0.379), glutamine at 0.533 mmol/L (IQR: 0.474–0.591), glycine at 0.139 mmol/L (IQR: 0.112–0.172), histidine at 0.064 mmol/L (IQR: 0.057–0.071), and valine at 0.227 mmol/L (IQR: 0.200–0.259). Complications associated with T2DM were relatively infrequent within this population. Specifically, renal complications were reported in only 156 patients (1.1%), while 1173 (8.3%) experienced ophthalmic issues, 353 (2.5%) had neurological complications, and 238 (1.7%) faced peripheral circulatory conditions. Notably, IR was prevalent, with 9010 patients (64.1%) exhibiting this condition (Table 2).

**Table 2:** Demographic information, amino acid levels, and related complications in patients with Type 2 diabetes.

**Variable**	**N** = 14,066^**a**^
Age (years)	60 (53, 65)
Sex	
Female	5710 (46.5%)
Male	8356 (53.5%)
Smoking	
No	6487 (46.5%)
Yes	7462 (53.5%)
Alcohol	
No	1202 (8.6%)
Yes	12,809 (91.4%)
Hypertension	
No	8348 (59.3%)
Yes	5718 (40.7%)
Body mass index	30.7 (27.6, 34.6)
Amino acid levels	
Alanine (mmol/L)	0.318 (0.265, 0.379)
Glutamine (mmol/L)	0.533 (0.474, 0.591)
Glycine (mmol/L)	0.139 (0.112, 0.172)
Histidine (mmol/L)	0.064 (0.057, 0.071)
Isoleucine (mmol/L)	0.054 (0.045, 0.067)
Leucine (mmol/L)	0.111 (0.094, 0.131)
Phenylalanine (mmol/L)	0.049 (0.042, 0.056)
Tyrosine (mmol/L)	0.066 (0.057, 0.076)
Valine (mmol/L)	0.227 (0.200, 0.259)
Complications/secondary outcomes	
Diabetes mellitus with renal complications	
No	13,910 (98.9%)
Yes	156 (1.1%)
Diabetes mellitus with ophthalmic complications	
No	12,893 (91.7%)
Yes	1173 (8.3%)
Diabetes mellitus with neurological complications	
No	13,713 (97.5%)
Yes	353 (2.5%)
Diabetes with peripheral circulatory complications	
No	13,828 (98.3%)
Yes	238 (1.7%)
Insulin use	
No	13,081 (93.0%)
Yes	985 (7.0%)
Oral hypoglycemic drug use	
No	11,106 (79.0%)
Yes	2960 (21.0%)
Insulin resistance	
No	5056 (35.9%)
Yes	9010 (64.1%)

^a^Mean ± SD; *n* (%).

### 3.5. The Predictive Role of AAs in the Development of Secondary Related Complications in Patients With T2DM

In the visual representation of predictive capabilities in Figure 5, it becomes evident that the prognostic value of AA indicators varies significantly across different secondary complications of diabetes. Specifically, for diabetes‐related renal complications, the aggregate AA profile, encompassing five distinct species, yields an AUC of 0.737 for the ROC curve. This AUC value suggests a predictive accuracy that exceeds the threshold for moderate reliability, indicating a robust association between the composite AA signature and the risk of renal complications in diabetic patients. When examining ophthalmic complications, the predictive AUC for the total AA profile is slightly lower at 0.731, yet still indicative of moderate accuracy. However, its discriminative power is somewhat less pronounced than for renal complications. In the case of neurological complications, the AUC for the total AA profile reaches 0.771, denoting a high predictive accuracy. This elevated AUC value implies that the overall AA profile is a strong predictor for neurological complications, potentially offering valuable insights for early identification and intervention. Lastly, for peripheral vascular complications, the AUC for the total AA profile is 0.732, which, akin to the ophthalmic complications, reflects a moderate level of predictive accuracy.

Figure 5ROC curve of AA levels and complications related to T2DM. (a) Neurological complications. (b) Ophthalmic complications. (c) Renal complications. (d) Peripheral circulatory complications. ROC, receiver operating characteristic; AUC, area under the curve.

**Figure:**
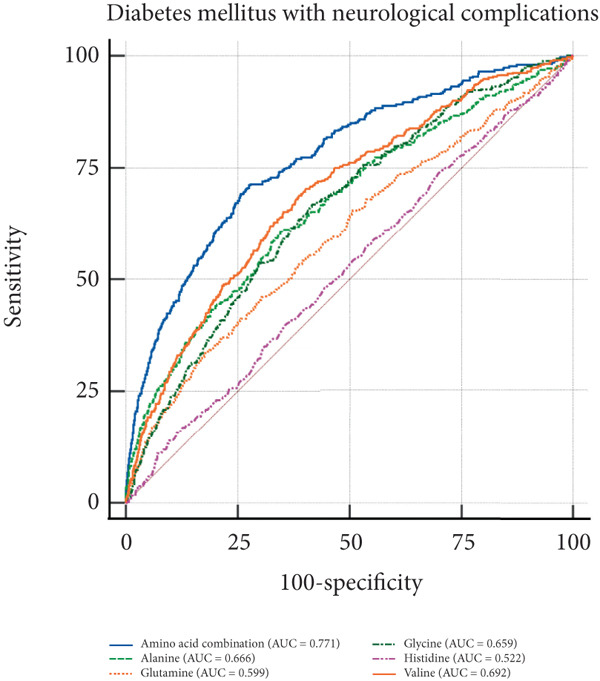
(a)

**Figure:**
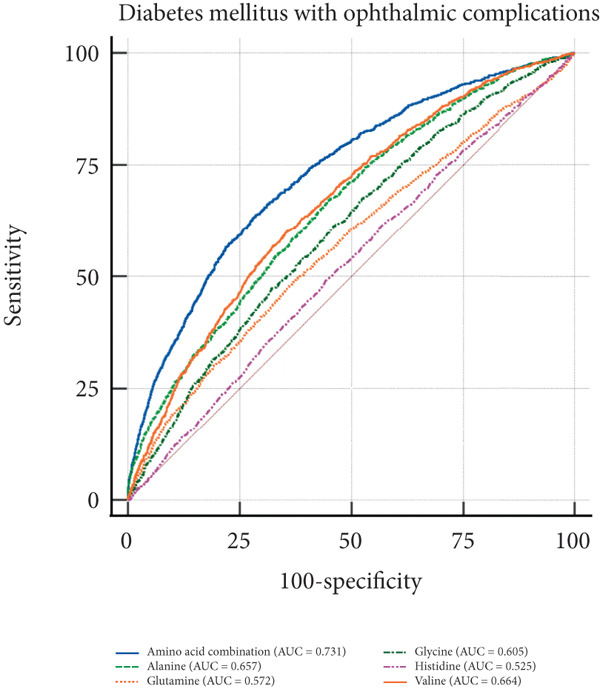
(b)

**Figure:**
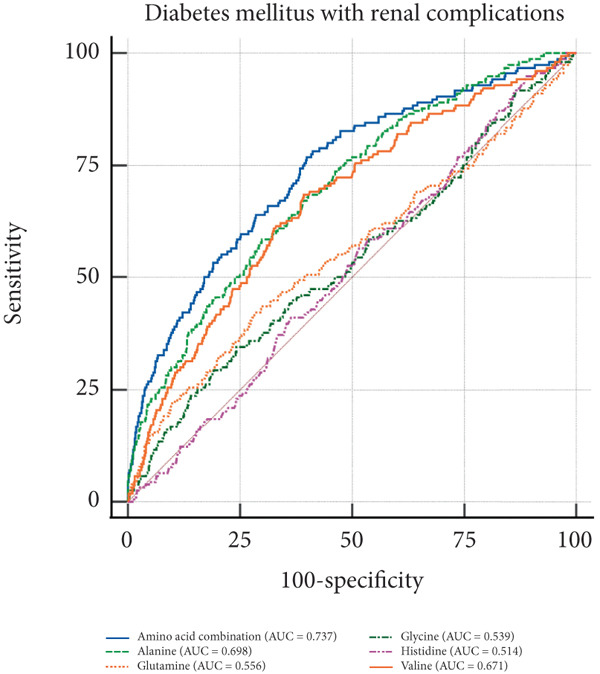
(c)

**Figure:**
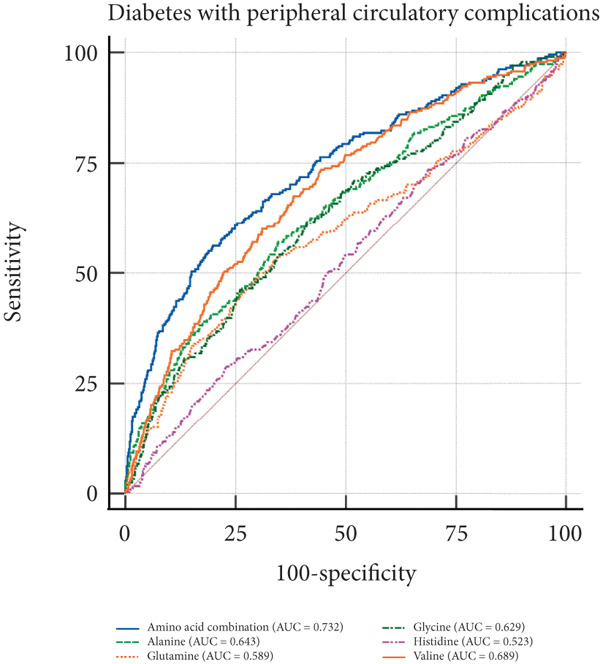
(d)

### 3.6. Correlation of AAs With IR

This study employed RCS analysis to investigate the association between AA levels and IR in patients with T2DM. As shown in Figure 6, a nonlinear relationship was observed between glutamine and glycine levels with IR (*p* nonlinear < 0.05). Specifically, when glutamine levels were below 0.60, each unit increase in glutamine concentration was associated with a 16% reduction in the risk of IR (OR = 0.84; 95% CI: 0.81–0.87, *p* < 0.001). Similarly, for glycine, when levels were under 0.205, each unit increase in glycine concentration corresponded to a 14% decrease in the risk of IR (OR = 0.86; 95% CI: 0.83–0.90, *p* < 0.001) (Table S2). In contrast, alanine and valine demonstrated a linear relationship with IR in the T2DM population (*p* nonlinear > 0.05), indicating that higher concentrations of these AAs were positively associated with an increased risk of IR. However, histidine levels showed no significant correlation with the occurrence of IR (*p* > 0.05).

Figure 6RCS curves of AA levels and IR in people with T2DM. (a) Alanine. (b) Glutamine. (c) Glycine. (d) Histidine. (e) Valine. RCS, restricted cubic spline; AA, amino acid; IR, insulin resistance; T2DM, Type 2 diabetes mellitus; 95% CI, 95% confidence interval.

**Figure:**
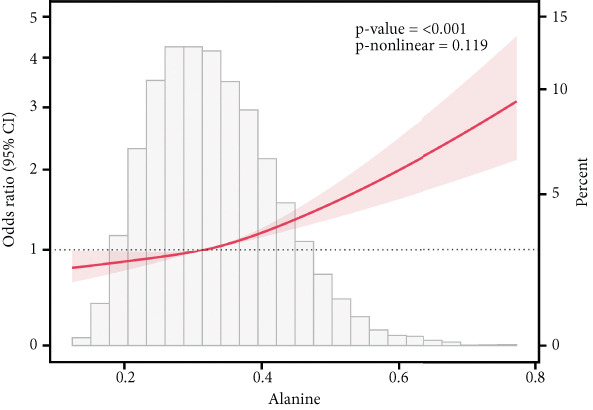
(a)

**Figure:**
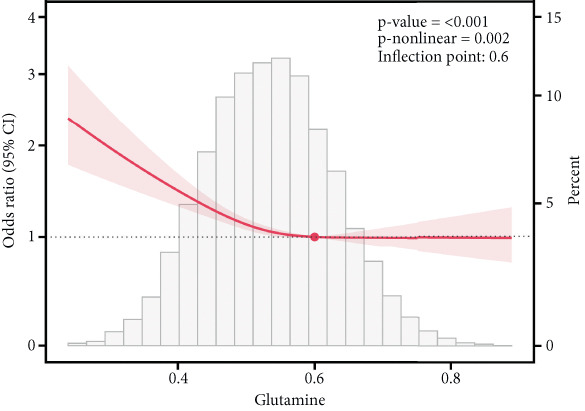
(b)

**Figure:**
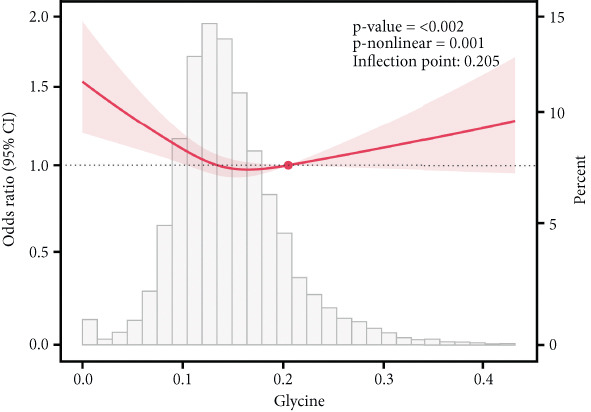
(c)

**Figure:**
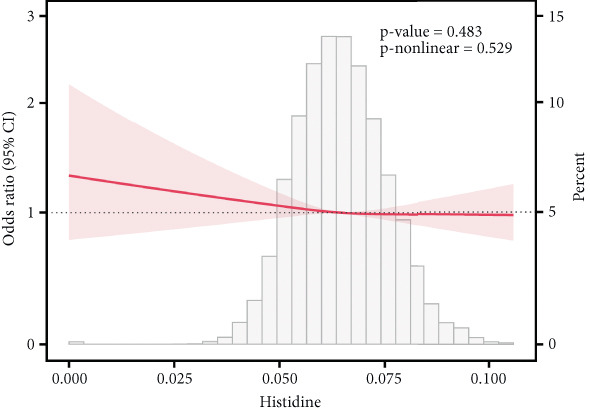
(d)

**Figure:**
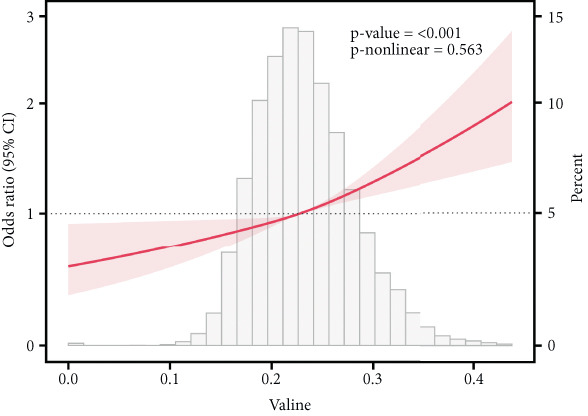
(e)

### 3.7. Correlation of AAs With Insulin Use

Our study investigates the correlation between AA metabolism and insulin dependence in patients with T2DM **(**Table 3 **)**. Among the findings, alanine showed a significant positive association with insulin use across all models. Specifically, after adjusting for sex, age, smoking, alcohol consumption, hypertension, and BMI (Model 4), each unit increase in alanine level was associated with a 31% higher likelihood of requiring insulin therapy (OR = 1.31, 95% CI: 1.24–1.38, *p* < 0.001). Histidine, in contrast, exhibited a significant negative association with insulin use, as observed in Model 4. Higher histidine levels were associated with a 7% reduction in the likelihood of insulin use (OR = 0.93, 95% CI: 0.86–0.99, *p* = 0.033). For other AAs, such as glutamine, glycine, and valine, no significant correlation with insulin use was found (*p* > 0.05), indicating that variations in their levels are not associated with insulin dependence in T2DM patients.

**Table 3:** Correlation between amino acid metabolism and insulin dependence.

**Amino Acid**	**Model 1**			**Model 2**			**Model 3**			**Model 4**		
	**OR**	**95% CI**	**p** **value**	**OR**	**95% CI**	**p** **value**	**OR**	**95% CI**	**p** **value**	**OR**	**95% CI**	**p** **value**
Alanine	1.31	1.25, 1.38	< 0.001	1.32	1.25, 1.39	< 0.001	1.31	1.24, 1.38	< 0.001	1.31	1.24, 1.38	< 0.001
Glutamine	1.05	0.99, 1.12	0.098	1.06	1.00, 1.13	0.06	1.06	1.00, 1.13	0.055	1.04	0.97, 1.10	0.254
Glycine	1.06	1.00, 1.13	0.066	1.06	1.00, 1.13	0.065	1.06	0.99, 1.13	0.092	1.05	0.99, 1.12	0.102
Histidine	0.95	0.88, 1.01	0.105	0.94	0.88, 1.01	0.076	0.94	0.88, 1.01	0.098	0.93	0.86, 0.99	0.033
Valine	0.99	0.93, 1.05	0.705	0.98	0.92, 1.04	0.513	0.97	0.91, 1.03	0.36	0.99	0.93, 1.06	0.797

*Note:* Model 1: no covariates were adjusted. Model 2: adjusted for sex and age. Model 3: adjusted for sex, age, smoking, alcohol, and hypertension. Model 4: adjusted for sex, age, smoking, alcohol, hypertension, and BMI.

Abbreviations: CI = confidence interval, OR = odds ratio.

### 3.8. Correlation of AAs With Oral Hypoglycemic Drug Use

Our study examined the relationship between AA levels and the use of oral hypoglycemic drugs in patients with T2DM, with results adjusted for confounding variables across different models (Table 4). Significant associations (*p* < 0.05) were identified for several AAs. Alanine demonstrated a strong positive correlation with oral hypoglycemic drug use across all models. Specifically, in the fully adjusted model (Model 4), each unit increase in alanine was associated with an 87% higher likelihood of requiring oral hypoglycemic drugs (OR = 1.87, 95% CI: 1.79–1.96, *p* < 0.001). Conversely, glutamine was significantly and inversely related to the use of oral hypoglycemic drugs, with each unit increase in glutamine resulting in a 15% lower likelihood of drug use (OR = 0.85, 95% CI: 0.82–0.89, *p* < 0.001) (Model 4). Similarly, glycine showed a modest but significant inverse correlation, with each unit increase reducing the likelihood of drug use by 6% (OR = 0.94, 95% CI: 0.90–0.98, *p* = 0.006). Histidine exhibited a borderline association with drug use, showing a significant negative correlation in Models 1–3 (*p* < 0.05). However, after full adjustment (Model 4), the relationship was no longer statistically significant (OR = 0.96, 95% CI: 0.92–1.01, *p* = 0.096). Valine, on the other hand, demonstrated a consistently strong positive association with oral hypoglycemic drug use. In the fully adjusted model (Model 4), each unit increase in valine was associated with a 27% higher likelihood of requiring oral hypoglycemic drugs (OR = 1.27, 95% CI: 1.22–1.32, *p* < 0.001).

**Table 4:** Correlation between amino acid metabolism and oral hypoglycemic drugs.

**Amino Acid**	**Model 1**			**Model 2**			**Model 3**			**Model 4**		
	**OR**	**95% CI**	**p** **value**	**OR**	**95% CI**	**p** **value**	**OR**	**95% CI**	**p** **value**	**OR**	**95% CI**	**p** **value**
Alanine	1.88	1.80, 1.96	< 0.001	1.87	1.80, 1.95	< 0.001	1.87	1.79, 1.95	< 0.001	1.87	1.79, 1.96	< 0.001
Glutamine	0.85	0.81, 0.88	< 0.001	0.83	0.80, 0.87	< 0.001	0.84	0.80, 0.87	< 0.001	0.85	0.82, 0.89	< 0.001
Glycine	0.95	0.91, 0.99	0.009	0.94	0.91, 0.98	0.007	0.94	0.90, 0.98	0.006	0.94	0.90, 0.98	0.006
Histidine	0.96	0.92, 1.00	0.042	0.95	0.91, 0.99	0.012	0.95	0.91, 1.00	0.035	0.96	0.92, 1.01	0.096
Valine	1.3	1.25, 1.35	< 0.001	1.29	1.24, 1.35	< 0.001	1.29	1.24, 1.35	< 0.001	1.27	1.22, 1.32	< 0.001

*Note:* Model 1: no covariates were adjusted. Model 2: adjusted for sex and age. Model 3: adjusted for sex, age, smoking, alcohol, and hypertension. Model 4: adjusted for sex, age, smoking, alcohol, hypertension, and BMI.

Abbreviations: CI = confidence interval, OR = odds ratio.

## 4. Discussion

T2DM is a systemic metabolic disease which not only has impaired glucose metabolism but also involved other metabolites alteration. By analyzing AA profile in a prospective cohort of UKB, we identified critical AAs associated with T2DM incidence, which were alanine, valine, glutamine, histidine, and glycine. And MR analysis showed that glycine, leucine, and glutamine are protective factors for T2DM, with OR of 0.881, 0.630, and 0.961, respectively (*p* < 0.05), indicating that these AAs are associated with a reduced risk of T2DM. Importantly, we performed ROC curve analysis and found that combination of these five AAs had shown good value in predicting secondary complications of T2DM, with all AUC > 0.73. These results suggest the AAs are valuable biomarkers during the onset and development of T2DM, which may aid in early diagnosis and dynamic follow‐up in clinical practice.

In recent years, previous studies, including case‐cohort analyses within randomized controlled trials such as the PREDIMED trial (Ruiz‐Canela et al. [2018]) and prospective cohort studies in young adults like the Cardiovascular Risk in Young Finns Study (Würtz et al. [2013]), have shown that elevated branched‐chain and aromatic AAs are associated with IR and increased risk of T2DM [25, 26]. And the Framingham Offspring Study found that all of the BCAA including isoleucine, leucine, and valine were positively associated with T2DM [27], which was consistent with the EPIC Potsdam study [28]. However, we only found a similar association between valine and T2DM in our study. Although isoleucine and leucine were also increased in the T2DM population compared with the nondiabetes population, the LASSO logistic regression analysis did not show a significant correlation between T2DM. This may be due to the differences in detection methods between the two, and magnetic resonance technology may encounter peak overlap issues when detecting metabolites, making it difficult to accurately identify and quantify some metabolites [29]. Instead, we found that alanine was another risk factor, whereas glutamine, histidine, and glycine were protective factors for T2DM (Figure 3b). Interestingly, Wang et al. conducted a nested case‐control study of 3414 incident T2DM cases and found that alanine was also a risk factor for incident T2DM, which was consistent with our findings [12]. Similarly, Vangipurapu et al. conducted the METSIM study of 5181 participants and found that alanine was associated with decreases in insulin secretion and an increased risk of incident T2DM [30]. Taken together, these results demonstrate that elevated alanine and valine were important risk factors for T2DM or incident T2DM. It will be very interesting to monitor the dynamic change of alanine and valine at the normoglycemic stage for the population with high risk of T2DM, as well as further evaluate the sensitivity and specificity for alanine and valine.

Glutamine and histidine have emerged as potential protective factors in the prevention of the development of T2DM [31–33]. According to prospective studies by Vangipurapu et al. and Wittenbecher et al., glutamine has been found to be negatively correlated with the occurrence and progression of T2DM, indicating its significant role in mitigating the onset of the disease [30, 34]. Furthermore, Welsh et al. have discovered that histidine levels are associated with a lower risk of mortality in patients with T2DM (OR = 0.89, 95% CI: 0.81–0.99, *p* = 0.024) [35]. Additionally, after oral administration of histidine, an increase in fasting plasma histidine concentration leads to various beneficial effects on glycemic control in T2D participants, including reduced fasting glucose, HbA1c, time in range, and average amplitude of glycemic excursions, as well as enhanced insulin sensitivity [36]. The mechanisms behind this protective effect may involve the modulation of inflammatory responses. It is widely accepted that inflammatory responses lead to IR, which is one of the significant hallmarks of T2DM [37]. Glutamine has been shown to exert anti‐inflammatory effects by regulating the production of proinflammatory cytokines, potentially reducing the inflammatory burden on pancreatic *β*‐cells and preserving their insulin‐secreting capacity [38]. On the other hand, histidine may contribute to maintaining cellular redox balance and preventing oxidative stress‐induced cellular damage, which is another critical factor in the pathogenesis of T2DM [37].

Our study confirmed that older age, higher BMI, hypertension, and smoking are established risk factors for T2DM, consistent with prior research. Interestingly, we found that male sex was linked to an increased risk of T2DM, while alcohol consumption showed a protective association. The higher risk in men may stem from differences in visceral fat distribution, sex hormone levels—such as lower estrogen and higher androgens—and lifestyle factors including higher smoking rates and less physical activity [39]. One possible explanation for the protective effect of alcohol is that moderate intake may improve insulin sensitivity, reduce systemic inflammation, and regulate hepatic glucose metabolism [40, 41]. Another plausible mechanism is that alcohol may interfere with AA absorption, which in turn reduces circulating AA levels and lowers susceptibility to T2DM [42].

Several studies have explored the link between plasma AAs and the complications or outcomes of T2DM. Moreno‐Vedia et al. conducted a prospective study involving 307 patients with T2DM or other metabolic disorders. Over a 10‐year follow‐up period, they observed that higher baseline levels of valine and alanine were significantly associated with an increased risk of developing atherosclerotic cardiovascular disease (ASCVD) in individuals with T2DM [17]. Consistently, we demonstrated a positive correlation between alanine, valine, and T2DM complications in our study. And glutamine and histidine were inversely associated with T2DM complications. Indeed, the combination of these five AAs could achieve better predictive ability compared with a single AA. Notably, the ADVANCE trial by Welsh et al. showed distinct associations between circulating AAs and risk of different complications of T2DM [35]. For example, they found that histidine was inversely associated with macrovascular disease but was not associated with microvascular disease. Unexpectedly, higher valine levels were associated with a lower risk of all‐cause mortality. These results indicate that there may be heterogeneity in terms of the association between circulating AAs and different T2DM complications. Future studies with designation of a large cohort, long‐term follow‐up, and repeated monitoring of circulating AAs are needed to further validate our and other investigators’ findings.

Although our findings indicate that specific AA levels were associated with T2DM and its related complications, the therapeutic efficacy by modulating or reducing specific AA level for preventing T2DM is not investigated. Importantly, some possible side effects should be considered before clinical application. Firstly, the reduction of BCAAs, which are indispensable for muscle protein synthesis and preservation, may inadvertently exacerbate sarcopenia, especially among elderly or chronically ill patients [43]. Secondly, AAs like glutamine play critical roles in maintaining immune function and intestinal barrier integrity; thus, their depletion could compromise immune responses and gastrointestinal permeability disorders [44]. Consequently, any intervention aimed at altering AA metabolism must carefully balance the anticipated metabolic benefits against these potential adverse effects. Rigorous clinical trials are essential to comprehensively evaluate both efficacy and safety, with particular attention to muscle integrity, immune competence, and overall metabolic homeostasis.

Several limitations must be acknowledged. Although our study supports a potential causal role of AAs in T2DM development, the disease is shaped by complex interactions among genetic, environmental, and epigenetic factors. The SNP‐based instruments used may not fully capture gene–gene, gene–environment interactions, or dynamic epigenetic modifications. Future studies integrating GWAS, epigenomics, and longitudinal data are needed to better elucidate these mechanisms. Moreover, while we applied stringent LD pruning criteria (*r* ^2^ < 0.001 and > 10,000 kb) to ensure the independence of instrumental variables, this conservative approach may have inadvertently excluded SNPs in moderate linkage that carry biological relevance, potentially introducing imprecision in identifying the full spectrum of causal variants. Additionally, the cross‐sectional nature of AA measurements may not capture long‐term metabolic changes, and the complexity of T2DM’s pathophysiology may not be fully represented by single nutrient analyses. These limitations underscore the need for cautious interpretation of results and highlight the importance of further research to corroborate and expand upon these findings.

## 5. Conclusion

AA metabolism, often an overlooked aspect in diabetes research, holds significant implications for the diagnosis, treatment, and prediction of complications in T2DM. The current use of large‐scale databases, such as the UKB, complements the limitations of previous smaller prospective studies by providing a broader and more diverse cohort. This approach strengthens the evidence for the role of AA levels in T2DM and underscores the importance of considering AA metabolism in clinical practice. A thorough understanding of AA metabolism allows us to implement more targeted therapeutic interventions for the T2DM population. Furthermore, it aids in the early identification of diabetes risk and complications, enabling preventive measures to be taken, thereby reducing the societal burden of this chronic disease.

## Ethics Statement

UK Biobank has approval from the North West Multicentre Research Ethics Committee (MREC) (REC reference: 21/NW/0157) as a Research Tissue Bank (RTB) approval. This approval means that researchers do not require separate ethical clearance and can operate under the RTB approval. The UK Biobank Ethics and Governance Council (EGC) informed consent was acquired from the patient(s) or a relative/guardian) and that the patient(s) (or relative/guardian) consented to the publishing of all images, clinical data, and other data included in the manuscript.

## Conflicts of Interest

The authors declare no conflicts of interest.

## Author Contributions

Jiayu Yang, Zelong Wu, Jike Fang, Sheng Chen, Baohua Hou, Chuanzhao Zhang, and Shanzhou Huang conceived and designed the study. Jiayu Yang, Shiye Ruan, Zhongyan Zhang, and Jinwei Cui were responsible for data collection. Jiayu Yang, Shiye Ruan, Zhongyan Zhang, and Qian Yan performed data analysis. Jiayu Yang, Jiangyuan Huang, Yajie Wang, and Jike Fang drafted the manuscript. Jiayu Yang, Shiye Ruan, Zelong Wu, and Qianlong Wu created the figures. Sheng Chen, Baohua Hou, Chuanzhao Zhang, and Shanzhou Huang reviewed, revised, and approved the article before submission. Shanzhou Huang is the guarantor of this work and, as such, has full access to all the data in the study and takes responsibility for the integrity and accuracy of the data analysis. Jiayu Yang, Zelong Wu, and Jike Fang contributed equally to this work.

## Funding

This work was supported by Wu Jieping Medical Foundation (320.6750.2025‐18‐11) and the Science and Technology Program of Maoming (2024kjcxLX046).

## Supporting Information

Additional supporting information can be found online in the Supporting Information section.

## Supporting information


**Supporting Information 1** Supplementary Figures Figure S1: Scatter plot, funnel plot, forest plot, and leave‐one‐out plot of two‐sample Mendelian randomization of 20 common amino acids and Type 2 diabetes. (a–c) Scatter plot. (d–f) Funnel plot. (g–i) Forest plot. (j–l) Leave‐one‐out plot.

**Supporting Information 2** Supplementary Materials: Results of a two‐sample Mendelian randomization study of amino acids and diabetes mellitus.

**Supporting Information 3** Table S1: The mean decrease accuracy and mean decrease Gini of the random forest algorithm.

**Supporting Information 4** Table S2: Table of the inflection points of the RCS curve of each amino acid level and insulin resistance.

## Data Availability

The data used to support the findings of this study are available from the corresponding authors upon request.
