# Supplementary material for: The Association Between Amino Acids and the Onset and Progression of Type 2 Diabetes Mellitus: A Comprehensive Analysis Based on UK Biobank Database
Source: J Diabetes Res. 2026 Jan 12;2026:8033429. doi: 10.1155/jdr/8033429 (PMC12794270; doi:10.1155/jdr/8033429)
Supplement: Supplementary file 1 — Supporting Information 1 Supplementary Figures Figure S1: Scatter plot, funnel plot, forest plot, and leave‐one‐out plot of two‐sample Mendelian randomization of 20 common amino acids and Type 2 diabetes. (a–c) Scatter plot. (d–f) Funnel plot. (g–i) Forest plot. (j–l) Leave‐one‐out plot. [file JDR-2026-8033429-s001.docx]

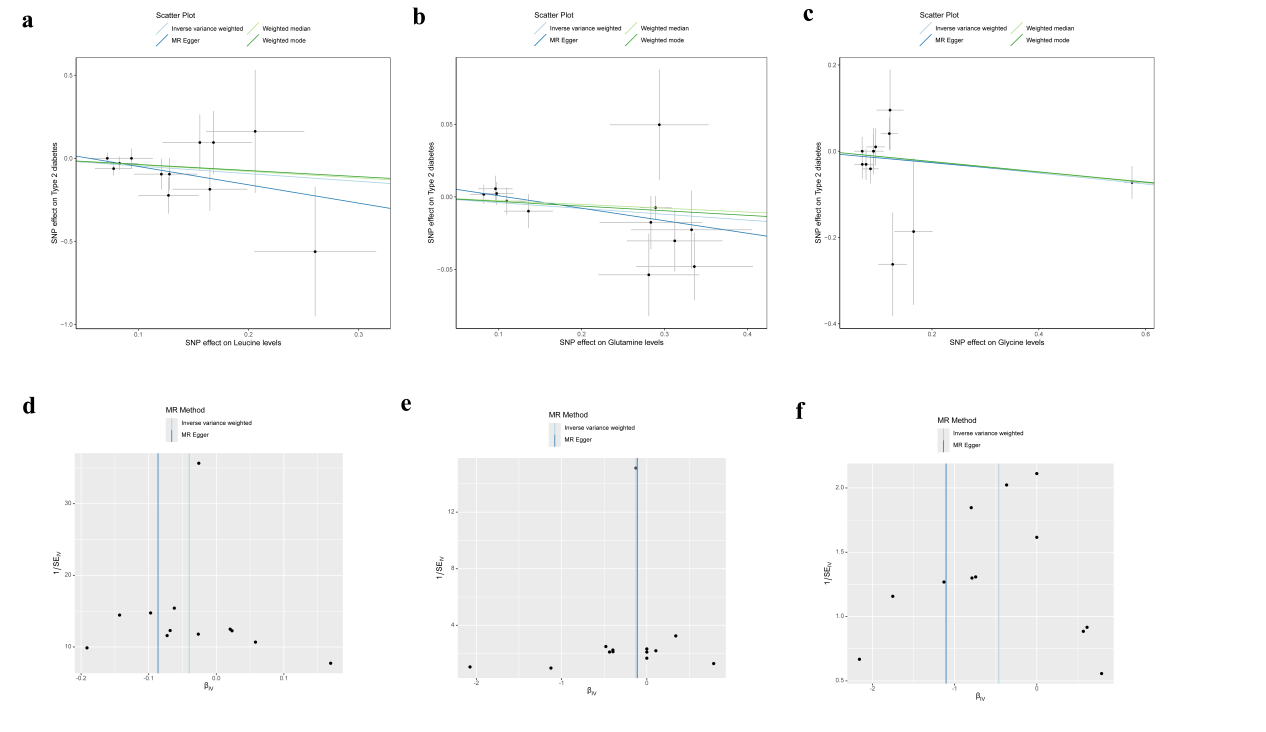


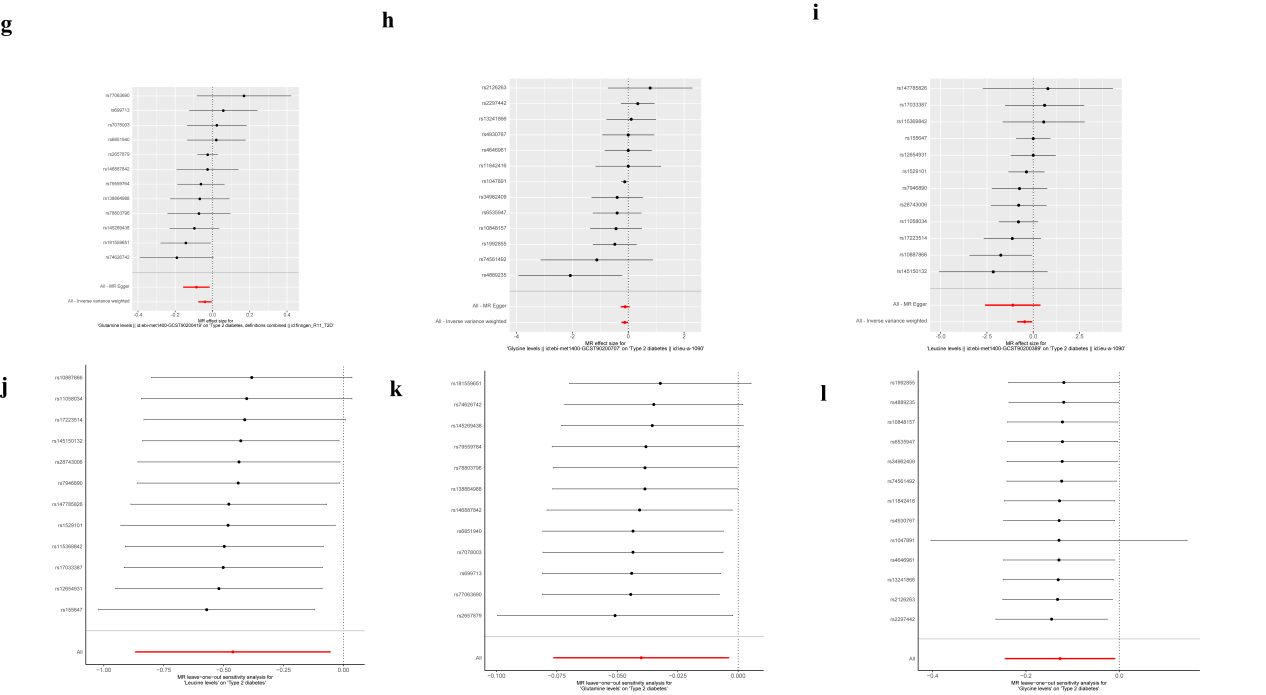


**Figure S1: Scatter plot, funnel plot, forest plot and leave-one-out plot of two-sample Mendelian randomization of 20 common amino acids and type 2 diabetes.** a-c:Scatter plot; d-f: funnel plot; g-i: forest plot; j-l: leave-one-out plot.
