# Supplementary material for: The Association Between Amino Acids and the Onset and Progression of Type 2 Diabetes Mellitus: A Comprehensive Analysis Based on UK Biobank Database
Source: J Diabetes Res. 2026 Jan 12;2026:8033429. doi: 10.1155/jdr/8033429 (PMC12794270; doi:10.1155/jdr/8033429)
Supplement: Supplementary file 3 — Supporting Information 3 Table S1: The mean decrease accuracy and mean decrease Gini of the random forest algorithm. [file JDR-2026-8033429-s003.docx]

**Table S1: The Mean Decrease Accuracy and Mean Decrease Gini of the random forest algorithm.**

| vars | Mean Decrease Accuracy | Mean Decrease Gini |
| --- | --- | --- |
| Tyrosine | 136.511876 | 2,166.9870 |
| Phenylalanine | 105.657198 | 2,039.5425 |
| Valine | 98.569104 | 2,325.3660 |
| Isoleucine | 91.569589 | 2,057.2942 |
| Leucine | 86.702127 | 2,111.7117 |
| Body.mass.index | 68.090638 | 2,987.6125 |
| Histidine | 63.441638 | 2,107.1705 |
| Glutamine | 61.193877 | 2,335.9060 |
| Alanine | 59.495769 | 2,468.8456 |
| Sex | 50.877185 | 232.9892 |
| Glycine | 49.894833 | 2,307.4591 |
| Age | 35.578460 | 1,544.6318 |
| Hypertension | 32.592615 | 427.6278 |
| Alcohol | 29.453154 | 187.8569 |
| Smoking | 2.487054 | 247.8002 |
