# Supplementary material for: The Association Between Amino Acids and the Onset and Progression of Type 2 Diabetes Mellitus: A Comprehensive Analysis Based on UK Biobank Database
Source: J Diabetes Res. 2026 Jan 12;2026:8033429. doi: 10.1155/jdr/8033429 (PMC12794270; doi:10.1155/jdr/8033429)
Supplement: Supplementary file 4 — Supporting Information 4 Table S2: Table of the inflection points of the RCS curve of each amino acid level and insulin resistance. [file JDR-2026-8033429-s004.docx]

**Table S2**. Table of the inflection points of the RCS curve of each amino acid level and insulin resistance

| **Characteristic** | **OR per SD** | **95% CI** | **p-value** |
| --- | --- | --- | --- |
| **Glutamine** |  |  |  |
| < 0.60 | 0.84 | 0.81, 0.87 | <0.001 |
| ≥ 0.60 | 0.93 | 0.87, 1.00 | 0.055 |
| **Glycine** |  |  |  |
| < 0.205 | 0.86 | 0.83, 0.90 | <0.001 |
| ≥ 0.205 | 0.91 | 0.83, 1.01 | 0.069 |
